# Assessment and validation of spot urine in estimating the 24-h urinary sodium, potassium, and sodium/potassium ratio in Chinese adults

**DOI:** 10.1038/s41371-019-0274-z

**Published:** 2019-10-28

**Authors:** Jianwei Xu, Xiaofu Du, Yamin Bai, Le Fang, Min Liu, Ning Ji, Jieming Zhong, Min Yu, Jing Wu

**Affiliations:** 1 National Center for Chronic and Noncommunicable Disease Control and Prevention, Chinese Center for Disease Control and Prevention, Beijing, China; 2 Zhejiang Provincial Center for Disease Control and Prevention, Hangzhou, China

**Keywords:** Risk factors, Nutritional supplements

## Abstract

The commonly used methods of estimating the 24-h urinary sodium (UNa) and urinary potassium (UK) from spot urine (SU) are the Kawasaki method (K-method), INTERSALT method (I-method), and Tanaka method (T-method), but the method that is suitable for use in the general Chinese population is still uncertain. We aimed to assess and validate these methods in estimating the 24-h UNa and UK using SU samples in Chinese adults. We studied 1428 individuals aged 18–69 years using SU and 24-h urine samples. For the K-method, I-method, and T-method, the Pearson correlation coefficients of the 24-h UNa were 0.35, 0.35, and 0.33 (all *p* < 0.01), and the intraclass correlation coefficients (ICC) were 0.34, 0.26, and 0.26 (all *p* < 0.01), respectively. The estimated 24-h UK using the K-method and T-method had correlation coefficients of 0.36 and 0.39 (all *p* < 0.01) and ICCs of 0.31 and 0.27 (all *p* < 0.01). The mean bias for the K-method in estimating the 24-h UNa and UK were the least biased among these methods. The bias between the 24-h urine Na/K ratio and the spot urinary Na/K ratio by the Bland–Altman method was −0.22. These methods for estimating the 24-h UNa and UK from SU were inadequate at the population level in Zhejiang Province, although the K-method showed the least bias among these methods. The spot urine Na/K ratio may be a useful and alternative method for 24-h urine collection for the estimation of the urinary Na/K ratio in the Chinese population.

## Introduction

Excess sodium intake increases the risk for high blood pressure and is a leading risk factor for cardiovascular disease [1, 2]. Reducing dietary salt intake is a vital way to prevent cardiovascular disease and constitutes a potentially important target for the improvement of public health [3]. Monitoring the sodium intake of a population must be accompanied by public health initiatives targeting sodium reduction. Daily food consumption recalls and 24-h urine collection are two of the main methods for estimating daily sodium and potassium intake [4, 5]. However, dietary assessments are time consuming and underestimate the intake due to underreporting and difficulties in quantifying the sodium concentration in recipes and discretionary salt [5]. The process of 24-h urine collection is widely considered the ‘gold standard’ for assessing dietary sodium and potassium intake [5, 6].

However, 24-h urine collection is very inconvenient in large epidemiological surveys in free-living populations [4, 5]. Limitations of 24-h urinary estimates include high labour costs and low response rates, especially in people who are active and work in manual occupations or occupations that require travelling. These factors affect the practicality of using the test in public health checkups or in population epidemiological surveys. Therefore, simple, objective, and standardized methods for measuring sodium and potassium intake at the population level are essential [7].

Based on the above considerations, the spot urine (SU) method is an alternative in estimating daily urine sodium and potassium excretion that has attracted increasing attention. Several studies have developed equations for facile and precise 24-h urinary sodium (UNa) and urinary potassium (UK) estimation based on SU. The mostly commonly used methods are the Kawasaki method (K-method) [8], INTERSALT method (I-method) [9], and Tanaka method (T-method) [10]. The validity of all the formulas in diverse international populations with a wide range of sodium intake is unknown. These methods are not yet readily available for the Chinese population, and the validation of these three methods in the Chinese population is limited.

Some studies also recently found that the Na/K ratio from repeated casual urine specimens is useful for estimating the 24-h Na/K ratio in normotensive and hypertensive Japanese individuals [11, 12]. The INTERSALT study assessed SU to estimate the 24-h urinary Na/K ratio, and the study found that the SU Na/K ratio with an appropriate bias correction may be useful for estimating the 24-h urinary Na/K ratio across different populations [13]. These findings show that there is no problematic bias in the Na/K ratio [14]. However, the accuracy of the Na/K ratio in the Chinese population is unknown.

The aim of this study was to validate and assess these methods to predict the 24-h UNa, UK, and Na/K ratio using SU samples among the general Chinese population.

## Methods

### Study participants

This study is based on the data obtained from the baseline survey of the Zhejiang salt reduction and Hypertension project, which was conducted at five sites in Zhejiang Province in 2016. This cross-sectional survey used a four-stage stratified sampling method to select a representative sample of the general adult population aged 18–69 years. A total of 7500 participants aged 18–69 years were selected among five counties and districts. All participants were invited to participate in a close-ended questionnaire, and a physical examination was conducted by trained public health staff. A subsample of 1500 participants were randomly selected and invited to provide 24-h urine collections and morning SU.

Our study was conducted according to the Declaration of Helsinki guidelines and any procedures involving human subjects were approved by the ethics committee of the National Center for Chronic and Noncommunicable Disease Control and Prevention, Chinese Center for Disease Control and Prevention. All study participants provided written informed consent.

### Urine collection and analysis

Before the investigation began, health professionals suggested that the subjects should not change their daily life habits. The participants were given a 50 ml cup and a 10 ml tube to collect their morning fasting urine at home and carried the SU to the health professional. Then, the health professional carefully explained to the subjects the key point of the 24-h urine collection and asked the subjects to correctly repeat the process. The participants were given a standard plastic container containing ~1.0 g boric acid as a preservative for the 24-h urine. The first urine was discarded before collection. The supervising health professional recorded the start and end times of each 24-h urine collection and determined the exact duration of the collection.

Each participant was interviewed using a standard questionnaire to assess the completeness of the 24-h urine collection. The total volume of the collection was measured by a laboratory technician, and the urine aliquots were frozen at −20 °C and shipped to a certified laboratory (Kingmed Center for Clinical Co., Ltd. Hangzhou, China). UNa and UK were measured with an ion-selective electrode method using the Abbott Architect C16000 autoanalyzer. Urinary creatinine was measured with the picric acid method using the Cobas C501 analyzer. The completeness of 24-h urine collection was defined by urine volume and urinary creatinine. A 24-h urinary volume less than 500 ml or a 24-h urinary creatinine volume that was ±2 standard deviations (SD) outside of the sex-specific mean were considered incomplete [15].

### Other measurements

We collected the subjects' sociodemographic characteristics and several lifestyle factors using face-to-face interviews by specially trained research staff and employing a standard questionnaire. The physical measurements included height, weight, waist circumference, and blood pressure. The height and weight (without shoes) was obtained using standardized techniques and calibrated equipment. The body-mass index formula was as follows: weight (kg)/height (m)^2^. Blood pressure was measured three times by an electronic sphygmomanometer (HEM-7071, OMRON Healthcare, Japan), and the average of the three measures was calculated and used for the analyses.

### Estimation of 24-h UNa and UK

We used the K-method, I-method, and T-method to estimate 24-h UNa using the SU sample. The INTERSALT formula was not designed to estimate the UK excretion, and therefore, we used the K-method and T-method to estimate 24-h UK excretion. All of the formulas’ expressions are available in Table 1.

**Table 1:** The formulas for estimating 24-h UNa and UK from SU samples

Method	Urine sample	Formula expression
Sodium		
K-method	Second morning urine	16.3 × (Na_su_/Cr_su_ × 1/10 × PrCr_24 h_)^0.5^
		Male: PrCr_24 h_ = 7.39 × height + 15.12 × weight − 12.63 × age − 79.9
		Female: PrCr_24 h_ = 5.09 × height + 8.58 × weight − 4.72 × age − 74.95
I-method	Casual SU	Male: 4.10 × BMI + (0.46 × Na_su_ + 25.46) − 2.75 × Cr_su_ − 0.13 × K_su_ + 0.26 × age
		Female: 2.39 × BMI + (0.34 × Na_su_ + 5.07) − 2.16 × Cr_su_ − 0.09 × K_su_ + 2.35 × age − 0.03 × age^2^
T-method	Casual SU	21.98 × (Na_su_/Cr_su_ × 1/10 × PrCr_24 h_)^0.392^
		PrCr_24 h_ = 16.14 × height + 14.89 × weight − 2.04 × age − 2244.45
Potassium		
K-method	Second morning urine	7.2 × (K_su_/Cr_su_ × 1/10 × PrCr_24 h_)^0.5^
		Male: PrCr_24 h_ = 7.39 × height + 15.12 × weight − 12.63 × age − 79.9
		Female: PrCr_24 h_ = 5.09 × height + 8.58 × weight − 4.72 × age − 74.95
T-method	Casual SU	7.59 × (K_su_/Cr_su_ × 1/10 × PrCr_24 h_)^0.431^
		PrCr_24 h_ = 16.14 × height + 14.89 × weight − 2.04 × age − 2244.45

The units of concentration of Na_su_ and K_su_ were all mmol/L, Cr_su_ was mg/dL, and the unit of PrCr_24 h_ was mg/day. Weight and height were kg and cm

*PrCr*_24 h_ predicted 24-h urinary creatinine, *Na*_*su*_ spot urinary sodium, *K*_*su*_ spot urinary potassium, *Cr*_*su*_ spot urinary creatinine

### Statistical analysis

Data are expressed as either the means and SD for continuous variables or as percentages for categorical variables. The estimated 24-h UNa and UK were calculated using these formulas in Table 1. The differences were computed by the estimated values minus the measured value of 24-h UNa and UK. The differences between the measured and estimated 24-h UNa (UK) were tested by the paired *t*-test. We used the Pearson correlation coefficient and intraclass correlation coefficients (ICCs) to assess the three methods between estimated and measured 24-h UNa (UK) and visualized the results using scatter plots. The Bland–Altman method was used to evaluate the agreement between the estimated and measured 24-h UNa (UK) [16]. The Pearson correlation coefficient for the Na/K ratio was calculated to examine the correlation between the values for SU and the corresponding values for 24-h urine specimens. Agreement between the SU Na/K ratio and the 24-h urine Na/k ratio was also examined using the Bland–Altman method. A sensitivity analysis was conducted in participants not taking antihypertensive medications. All statistical analyses were performed with SAS 9.3 (SAS Institute Inc.). All tests were two-sided, and a *p* value < 0.05 was considered significant.

## Results

### Subject characteristics

We excluded six participants with missing physical examinations or other variables of interest and 66 participants with incomplete 24-h urine collection. Finally, 1428 participants were included in the final analysis. The characteristics of the subjects are shown by sex in Table 2. A total of 49% of the participants were male. The mean age was 46.72 years (SD = 14.05 years). The mean 24-h urine volume was 1447.70 mL. The mean 24-h sodium excretion was 167.19 mmol/day, the 24-h potassium excretion was 37.42 mmol/day, and the 24-h sodium/potassium ratio was 4.95.

**Table 2:** Personal characteristics of the study participants by gender

Characteristic	All (*n* = 1428)	Male (*n* = 701)	Female (*n* = 727)	*P* value
Age (years)	46.72 ± 14.05	46.68 ± 14.42	46.75 ± 13.69	0.9185
Weight (kg)	62.97 ± 10.96	67.64 ± 10.45	58.48 ± 9.46	<0.0001
Height (cm)	161.61 ± 8.10	167.07 ± 6.52	156.34 ± 5.59	<0.0001
BMI (kg/m^2^)	24.05 ± 3.41	24.20 ± 3.22	23.91 ± 3.57	0.109
Systolic blood pressure (mm Hg)	130.02 ± 19.51	133.57 ± 18.08	126.61 ± 20.22	<0.0001
Diastolic blood pressure (mm Hg)	80.04 ± 10.90	82.16 ± 10.64	78.00 ± 10.76	<0.0001
Spot urine				
Sodium concentration (mmol/L)	125.35 ± 49.98	126.02 ± 49.33	124.70 ± 50.63	0.6177
Potassium concentration (mmol/L)	32.47 ± 17.02	32.54 ± 17.67	32.41 ± 16.38	0.8881
Sodium/potassium ratio	4.73 ± 2.99	4.84 ± 3.14	4.62 ± 2.85	0.1679
Creatinine concentration (mmol/L)	12.53 ± 6.50	14.18 ± 6.70	10.93 ± 5.88	<0.0001
24-h urine				
24-h urine volume (mL)	1447.70 ± 448.98	1480.22 ± 465.74	1416.33 ± 430.22	0.0071
24-h sodium excretion (mmol/day)	167.19 ± 74.70	174.64 ± 78.52	160.00 ± 70.12	0.0002
24-h potassium excretion (mmol/day)	37.42 ± 17.35	36.59 ± 19.01	38.21 ± 15.55	0.0786
24-h sodium/potassium ratio	4.95 ± 2.44	5.39 ± 2.57	4.53 ± 2.24	<0.0001
24-h creatinine excretion (mmol/day)	9.54 ± 3.91	10.96 ± 4.20	8.17 ± 3.05	<0.0001

### Mean measured and estimated 24-h UNa and UK

The differences between the measured and estimated 24-h UNa are presented in Table 3. The K-method showed the smallest difference among the three methods, which was 16.90 mmol/day (95% CI: 12.94, 20.86 mmol/day). The estimation from the I-method showed the largest difference, and the mean difference was −33.35 mmol/day (95% CI: −37.00, −29.69 mmol/day). The mean estimated 24-h UNa was higher than the measured values using the K-method (*t* = 8.38, *p* < 0.01). However, the mean estimated 24-h UNa using the I-method and T-method was lower than the measured 24-h UNa (*t* = −16.78, *p* < 0.01; *t* = −12.77, *p* < 0.01).

**Table 3:** Validity of the three methods of measured versus estimated 24-h UNa

Variables	Measured	K-method	I-method	T-method
Mean (mmol/day)				
All	167.19 ± 74.70	184.09 ± 56.84	133.84 ± 33.91	143.12 ± 35.63
Male	174.64 ± 78.52	192.66 ± 59.04	151.56 ± 34.42	142.91 ± 35.61
Female	160.00 ± 70.12	175.82 ± 53.39	116.76 ± 22.88	143.32 ± 35.67
Range (mmol/day)	20.94–550.68	55.24–417.57	26.85–253.28	55.56–273.67
Mean difference (mmol/day, 95% CI)^a^	Reference	16.90 (12.94, 20.86)	−33.35 (−37.00, −29.69)	−24.07 (−27.77, −20.38)
Intraclass correlation coefficient (95% CI)^b^	Reference	0.34 (0.29, 0.39)	0.26 (0.22, 0.31)	0.26 (0.21, 0.31)
Pearson correlation coefficient^c^	Reference	0.35	0.35	0.33

^a^Mean difference was calculated by the estimated 24-h UNa minus measured values and all *p* < 0.01; 95% CI: 95% confidence interval

^b^We used the value of the single measures and all *p*  <  0.01

^c^All *p* <   0.01

The differences between measured and estimated 24-h UK are presented in Table 4. The K-method showed the smallest difference between the two methods, which was 2.36 mmol/day (95% CI: 1.50, 3.21 mmol/day). The mean estimated 24-h UK was higher than the measured values using the K-method (*t* = 5.40, *p* < 0.01). However, the mean estimated 24-h UK using the T-method was lower than the measured 24-h UK (*t* = −12.10, *p* < 0.01).

**Table 4:** Validity of the two methods of measured versus estimated 24-h UK

Variables	Measured	K-method	T-method
Mean (mmol/day)			
All	37.42 ± 17.35	39.77 ± 9.70	32.29 ± 6.91
Male	36.59 ± 19.01	41.44 ± 10.34	32.09 ± 7.06
Female	38.21 ± 15.55	38.17 ± 8.75	32.49 ± 6.78
Range (mmol/day)	4.90–149.36	12.18–96.18	11.88–62.93
Mean difference (mmol/day, 95% CI)^a^	Reference	2.36 (1.50, 3.21)	−5.12 (−5.95, −4.29)
Intraclass correlation coefficient (95% CI)^b^	Reference	0.31 (0.26, 0.36)	0.27 (0.22, 0.31)
Pearson correlation coefficient^c^	Reference	0.36	0.39

^a^Mean difference was calculated by the estimated 24-h UK minus the measured values and all *p* < 0.01; 95% CI: 95% confidence interval

^b^We used the value of the single measures and all *p* < 0.01

^c^All *p*   < 0.01

### Assessment between estimated and measured 24-h UNa and UK

**Fig. 1:**
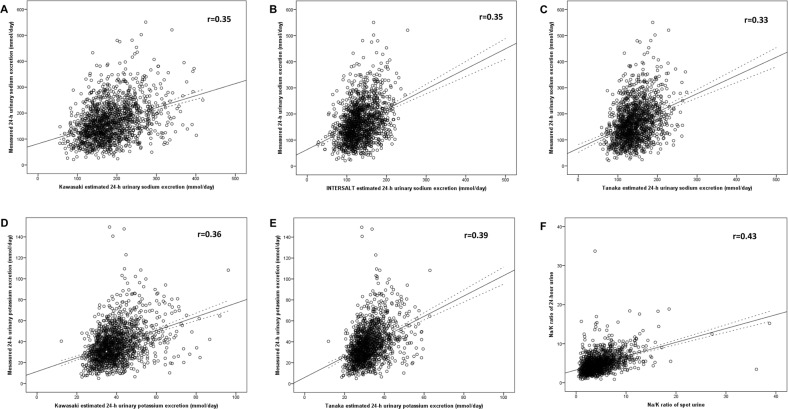
Scatter plots measured 24-h UNa vs. K-method (**a**), I-method (**b**), and T-method (**c**) methods estimated 24-h UNa, measured 24-h UK vs. K-method (**d**) and T-method (**e**) methods estimated 24-h UK, Na/K ratio of spot urine vs. 24-h urine (**f**). The dash lines were the 95% CI lines of predicted mean. The real line was the liner regression line

The Pearson correlation coefficients between the estimated and measured 24-h UNa using the three methods were all low (Fig. 1). The K-method and I-method had a Pearson correlation coefficient of 0.35, and the T-method had a Pearson correlation coefficient of 0.33 (all *p* < 0.01) (Fig. 1a–c). The Pearson correlation coefficients between the estimated and measured 24-h UK using the two methods were 0.36 for the K-method and 0.39 for the T-method (all *p* < 0.01) (Fig. 1d, e). The ICCs of the 24-h UNa were also low and were 0.34 for the K-method and 0.26 for the I-method and T-method (all *p* < 0.01). The ICCs of 24-h UK were 0.31 for the K-method and 0.27 for the T-method (all *p* < 0.01).

**Fig. 2:**
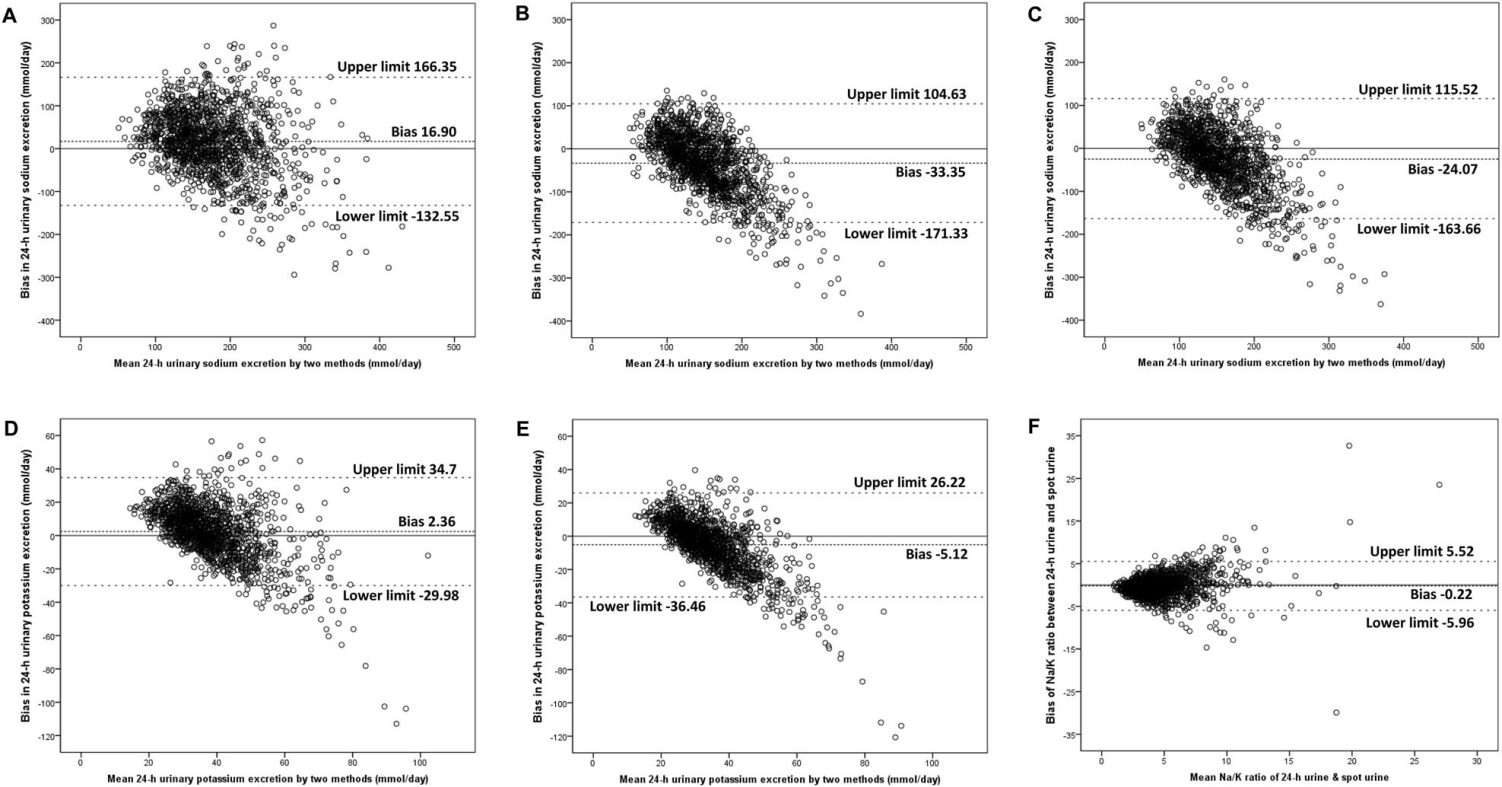
Bland–Altman plots presenting measured vs. estimated 24-h UNa using the K-method (**a**), I-method (**b**), and T-method (**c**), 24-h UK using the K-method (**d**) and T-method (**e**), Na/K ratio of spot urine vs. 24-h urine (**f**). The mid-dashed line was the mean difference. The upper and lower limits of agreement was the mean difference ± 1.96 × standard deviation

We used the Bland–Altman method to assess the agreement between the estimated and measured 24-h UNa (UK) (Fig. 2a–e). The results show a significant tendency of overestimation and underestimation depending on the bias. The mean estimated 24-h UNa and UK levels were consistently overestimated compared to the true measured values using the K-method and were consistently underestimated compared to the T-method. The I-method tendency of underestimated occurred for 24-h UNa. The smallest gap between estimated 24-h UNa (UK) and measured values were shown in the K-method, which was a relatively accurate method among these methods.

### Validation of the estimated 24-h urinary Na/K ratio from spot urine

The mean value of the Na/K ratio in SU was 4.73, and the mean value of the Na/K ratio in 24-h urine was 4.95. The spot urinary Na/K ratio was mostly lower than the 24-h urinary Na/K ratio (*P* < 0.01). The correlation coefficient for the Na/K ratio between the values for SU and the corresponding values for 24-h urine was 0.43 (Fig. 1f, *P* < 0.01), which was the highest compared with corresponding *R*-values for spot and 24-h UNa or UK (Tables 2 and 3). Using the Bland–Altman method, the bias between the 24-h urine Na/K ratio and the spot urinary Na/K ratio was −0.22 (95% CI: −5.96, 5.52) (Fig. 2f).

### Sensitivity analysis

Of the 1428 participants, 202 (14.1%) participants were taking an antihypertensive medication. After excluding the 202 individuals receiving antihypertensive medication, the results were similar (see Table S1–S3, Figs. S1 and S2 in supplementary material).

## Discussion

The K-method, I-method, and T-method are most commonly used to estimate the 24-h UNa and UK using SU samples. This study validated and assessed these methods in Chinese adults and assessed SU to estimate the 24-h urinary Na/K ratio. Our results showed that the K-method was the least biased among these methods based on the estimation of 24-h UNa and UK in Zhejiang Province. The most disappointing result is that overestimation or underestimation occurred in all three methods. However, using SU to estimate the 24-h Na/K ratio may be a more useful and alternative method.

We adjusted the process based on the creatinine concentration in all three methods because of its consistent excretion in urine [17–19]. If using SU to estimate 24-h urine collection as a substitution of a method, the agreement tests between the two methods need to be accepted. However, fairly low correlations between the estimated and measured sodium excretion were shown for all three methods in our study (*r* = 0.33–0.35, all *p* < 0.01; ICCs = 0.26–0.34, all *p* < 0.01). Compared with the results of previous studies, our results are similar or much lower [9, 20–23]. For example, the results of the PURE study showed K-method, I-method, and T-method correlation coefficients of 0.19, 0.19, and 0.29, respectively. Their ICCs ranged from 0.21 to 0.29, which was moderated [9]. Our results are similar with a study in Shaanxi Province in China, which reported correlation coefficients ranging from 0.35 to 0.38 and ICCs ranging from 0.31 to 0.38 [24]. Accordingly, a study on Chinese adults showed correlation coefficients that ranged from 0.18 to 0.29, and ICCs that were moderated from 0.21 to 0.29 [25]. Our study showed low correlation between the predicted and measured 24-h UNa, which indicated that the three methods may not be viable for individual sodium excretion estimations.

Many studies have suggested using the SU sample to estimate 24-h UNa as a substitute for the 24-h urine collection method [26]. However, the correlation analysis and Bland–Altman method should be performed to determine the agreement between the two methods before we apply the SU collection method in a large survey [16]. Our study showed that using the K-method to estimate 24-h UNa and UK from SU was the least biased. Similarly, the PURE study also showed that estimation of 24-h UNa using the K-method to be the least biased [7]. However, Cogswell et al. recently found that the I-method may provide the least biased estimation of 24-h UNa among young adults in the USA [27]. Bianca et al. also recently found that the estimations of Na excretion by the three formulas should be used with caution when reporting Na intake levels [28]. Polonia et al. found a poor agreement between the estimated and observed measurements of UNa and UK in a large national representative population [29].

Our study was similar to certain other studies in Chinese adults. The study in Shaanxi Province, China, also found significant biases for these three methods in estimating 24-h UNa, and the least bias and highest agreement occurred for the K-method (mean bias: 31.90 mmol/day, 95% Cl: 23.84, 39.97 mmol/day) [24]. Another study in Chinese adults showed that the K-method was the most acute estimate (mean bias −32.17 mmol/day, 95% Cl: −53, 11.39 mmol/day), but it underestimated the 24-h UNa presented in all three methods [25]. Another study in China showed that the K-method presented a more accurate estimation in Chinese hypertension patients [30]. However, so far, no confirmatory results have been found among the three methods to estimate 24-h UNa using SU in the Chinese population. Our study found that an overestimation tendency occurred for the K-method in estimating 24-h UNa. In contrast, the I-method and T-method underestimated 24-h sodium excretion. This important inconformity may be one of the reasons that these methods cannot replace the 24-h urine collection method. Note that the results in our current study showed underestimation or overestimation throughout low to high 24-h UNa levels. However, the PURE study illustrated that overestimation might occur in the lower sodium excretion sodium level, while underestimation might occur in the higher sodium level for the three methods [7]. The reason for the inconsistencies may be the different personal characteristics of the study participants.

Our study results reconfirmed that using SU to estimate 24-h UNa, the K-method, I-method, and T-method were inadequate at the Chinese population level. Our validation and assessment in Zhejiang Province showed a low accuracy for these common methods. One of the important reasons for this low accuracy may be the dietary lifestyle difference. In general, the K-method and T-method were based on the Japanese population, and the I-method was based on the European and North American population. In brief, these methods were not specifically established for the Chinese population. When these formulas were applied in the Chinese population, the bias is foreseeable. Moreover, the K-method was established using a second morning urine sample. Considering the large sample-size investigation convenience, in our study, we just collected the first morning urine instead of the second morning urine to estimate 24-h UNa. Therefore, validation studies in different populations, especially in the Chinese population, would be required. However, it might be uneasy to establish this kind of method to estimate population mean sodium intake under diverse dietary lifestyle available among nationwide China.

Our study found that the correlation coefficient for the 24-h Na/K ratio and SU Na/K ratio was 0.43, and the bias between the Na/K ratio of 24-h urine and spot urinary Na/K ratio by the Bland–Altman method was −0.22. The correlation coefficient in East Asian individuals from the INTERSALT study was *r* = 0.64, and the bias was 0.65 [13]. The reason for the lower correlation coefficient in our study may be we just collected the first morning urine for investigation convenience. Iwahori et al. found that the Na/K ratio of the first morning urine did not show a strong correlation with that of 7-day 24-h urine [11]. Although the bias reflecting diurnal variability is exists in estimation of the Na/K ratio in casual urine specimen, the Na/K ratio in SU may also be a useful proxy for 24-h values if repeated measurements are available to reduce measurement error and increase precision [11, 12]. Therefore, our findings suggest that the SU Na/K ratio may be a useful and alternative method to 24-h urine collection for the estimation of the urinary Na/K ratio in the Chinese population.

High dietary sodium and low dietary potassium intakes still exists over the past four decades in China [31]. Reducing the Na/K ratio is essential for preventing hypertension and cardiovascular disease in China. Currently, there is no generally accepted recommended guideline for the Na/K ratio. Base on the previous studies, a urinary Na/K molar ratio of 1.0 may be a target level [14]. Measurement of the casual urine Na/K ratio also has the potential for providing prompt feedback to individuals using a self-monitoring device [32]. However, it seemed difficult for Chinese participants to achieve the target level of Na/K ratio recommended. Further research is needed to establish what levels of the urinary Na/K ratio correspond to 5 g/day of salt intake in Chinese population. Setting a goal for the Na/K ratio may be helpful to support efforts to reduce Na and increase K for individuals [33], which has important public health implications in China.

To our knowledge, one of the major strengths of our study is a relatively large sample of Chinese adults to assess and validate the three methods and to estimate 24-h UNa using SU samples (as a first assessment to estimate 24-h UK and the 24-h Na/K ratio). Nevertheless, our study had several limitations. First, a single 24-h urine sample was not sufficient to assess the between- and within-individual variation for individual sodium and potassium intakes. The precision might be improved by collecting multiple 24-h urine samples per person [34]. However, 24-h urine sample collection was difficult due to the time requirement and inconvenience. In addition, the 24-h urine collection response rate and completeness is difficult to guarantee, especially in large epidemiological studies. Second, personal creatinine excretion is often considered to be relatively stable, but creatinine varied daily because of dietary protein intake and physical activity [35]. Considering these sensitive factors, we repeatedly emphasized that subjects should not change their daily life during our survey; however, it is difficult to ensure participant compliance. Third, we excluded those participants who self-reported kidney disease, but we did not evaluate the renal function of the participants, which may result with bias [36]. Lastly, we only sampled one province in China. Therefore, we need to validate these methods in more large-scale epidemiological research in Chinese.

These methods to estimate 24-h UNa and UK from SU were inadequate at the population level in Zhejiang Province; however, the K-method showed the least bias among these methods. The SU Na/K ratio may be a useful and alternative method to 24-h urine collection for estimation of the urinary Na/K ratio in the Chinese population.

## Summary

### What is known about topic

- 24-h urine collection is essential for assessing UNa and UK but this is very inconvenient in large epidemiological surveys.
- 24-h UNa and UK estimation equations based on SU have been developed but validity of these equations in Chinese population is still uncertain.
- The accuracy of estimating 24-h urinary Na/K ratio from spot urinary Na/K ratio in the Chinese population in unknown.

### What this study adds

- Large sample size of Chinese adults to validate three methods in estimating 24-h UNa and UK using SU.
- These methods for estimating the 24-h UNa and UK from SU were inadequate.
- The spot urinary Na/K ratio may be a useful and alternative method for 24-h urine collection for the estimation of the urinary Na/K ratio in the Chinese population.

## Supplementary information

supplementary material
