# Supplementary material for: Assessment and validation of spot urine in estimating the 24-h urinary sodium, potassium, and sodium/potassium ratio in Chinese adults
Source: J Hum Hypertens. 2019 Oct 28;34(2):184–92. doi: 10.1038/s41371-019-0274-z (PMC7027967; doi:10.1038/s41371-019-0274-z)
Supplement: Supplementary file 1 — supplementary material [file 41371_2019_274_MOESM1_ESM.docx]

| **Table S1** Characteristics of study participants excluding antihypertensive medication | | | | |
| --- | --- | --- | --- | --- |
| **Characteristic** | **All (n=1226)** | **Male (n=605)** | **Female (n=621)** | **P value** |
| Age (years) | 44.80±13.92 | 44.76±14.26 | 44.84±13.59 | 0.9197 |
| Weight (kg) | 62.47±10.98 | 67.14±10.45 | 57.93±9.47 | <0.0001 |
| Height (cm) | 161.63±8.12 | 167.02±6.67 | 156.39±5.57 | <0.0001 |
| BMI (kg/m^2^) | 23.85±3.42 | 24.04±3.26 | 23.67±3.57 | 0.0549 |
| Systolic blood pressure (mm Hg) | 127.56±18.77 | 131.01±16.94 | 124.19±19.84 | <0.0001 |
| Diastolic blood pressure (mm Hg) | 79.05±10.71 | 81.07±10.31 | 77.07±10.74 | <0.0001 |
| Spot urine |  |  |  |  |
| Sodium concentration (mmol/L) | 126.46±50.52 | 127.17±50.49 | 125.76±50.58 | 0.6261 |
| Potassium concentration (mmol/L) | 32.95±17.37 | 33.09±18.01 | 32.81±16.75 | 0.7750 |
| Sodium/potassium ratio | 4.74±3.08 | 4.82±3.20 | 4.66±2.95 | 0.3664 |
| Creatinine concentration (mmol/L) | 12.89±6.52 | 14.53±6.69 | 11.29±5.94 | <0.0001 |
| 24-h urine |  |  |  |  |
| 24-h urine volume (mL) | 1433.56±440.67 | 1463.71±450.43 | 1404.19±429.29 | 0.0180 |
| 24-h sodium excretion (mmol/day) | 168.90±75.48 | 175.92±79.04 | 162.07±71.24 | 0.0013 |
| 24-h potassium excretion (mmol/day) | 37.29±17.41 | 36.57±19.28 | 38.00±15.35 | 0.1510 |
| 24-h sodium/potassium ratio | 5.03±2.51 | 5.46±2.62 | 4.62±2.33 | <0.0001 |
| 24-h creatinine excretion (mmol/day) | 9.61±3.92 | 10.97±4.22 | 8.27±3.08 | <0.0001 |

| **Table S2** Validity of the three methods of measured versus estimated 24-h UNa in participants excluding antihypertensive medication | | | | |
| --- | --- | --- | --- | --- |
| Variables | Measured | K-method | I-method | T-method |
| Mean (mmol/day) |  |  |  |  |
| All | 168.90±75.48 | 182.56±56.56 | 133.02±33.99 | 141.55±35.24 |
| Male | 175.92±79.04 | 191.47±58.74 | 149.91±35.03 | 141.47±35.19 |
| Female | 162.07±71.24 | 173.87±52.97 | 116.57±23.20 | 141.63±35.33 |
| Range (mmol/day) | 20.94-550.68 | 55.24-417.57 | 26.85- 253.28 | 55.56- 273.67 |
| Mean difference (mmol/day, 95% CI)^a^ | Reference | 13.65(9.38, 17.92) | -35.88(-39.85, -31.91) | -27.35(-31.35, -23.35) |
| Intraclass correlation coefficient (95% CI)^b^ | Reference | 0.35(0.30,0.40) | 0.27(0.22,0.32) | 0.27(0.21,0.32) |
| Pearson correlation coefficient^c^ | Reference | 0.36 | 0.36 | 0.35 |
| ^a^Mean difference was calculated by the estimated 24-h UNa minus measured values and all *p<*0.01; 95% CI: 95% confidence interval; ^b^ We used the value of the single measures and all *p<*0.01; ^c^ all *p<*0.01 | | | | |

| **Table S3** Validity of the two methods of measured versus estimated 24-h UK in participants excluding antihypertensive medication | | | |
| --- | --- | --- | --- |
| Variables | Measured | K-method | T-method |
| Mean (mmol/day) |  |  |  |
| All | 37.29±17.41 | 39.53±9.76 | 31.95±6.86 |
| Male | 36.57±19.28 | 41.39±10.51 | 31.87±7.09 |
| Female | 38.00±15.35 | 37.71±8.60 | 32.04±6.63 |
| Range (mmol/day) | 4.90-149.36 | 12.18-96.18 | 11.88-62.93 |
| Mean difference (mmol/day, 95% CI)^a^ | Reference | 2.23(1.30, 3.17) | -5.34(-6.24, -4.44) |
| Intraclass correlation coefficient (95% CI)^b^ | Reference | 0.31(0.25,0.36) | 0.26(0.21,0.31) |
| Pearson correlation coefficient^c^ | Reference | 0.36 | 0.38 |
| ^a^Mean difference was calculated by the estimated 24-h UK minus the measured values and all *p<*0.01; 95% CI: 95% confidence interval; ^b^ We used the value of the single measures and all *p<*0.01; ^c^ all *p<*0.01 | | | |

**Figure S1** Scatter plots measured 24-h UNa vs. K-method (A), I-method (B) and T-method(C) methods estimated 24-h UNa, measured 24-h UK vs. K-method (D) and T-method(E) methods estimated 24-h UK, Na/K ratio of spot urine vs. 24-h urine (F). The dash lines were the 95% CI lines of predicted mean. The real line was the liner regression line. (1226 participants excluding antihypertensive medication).


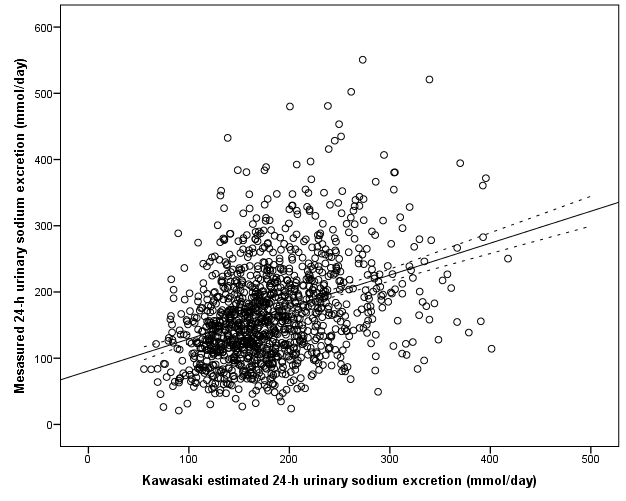

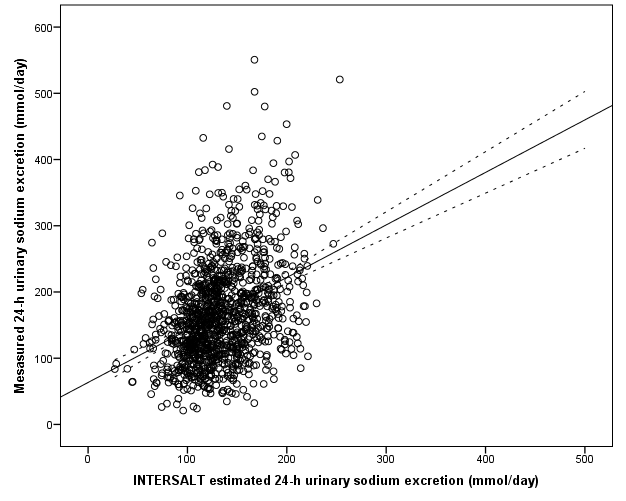

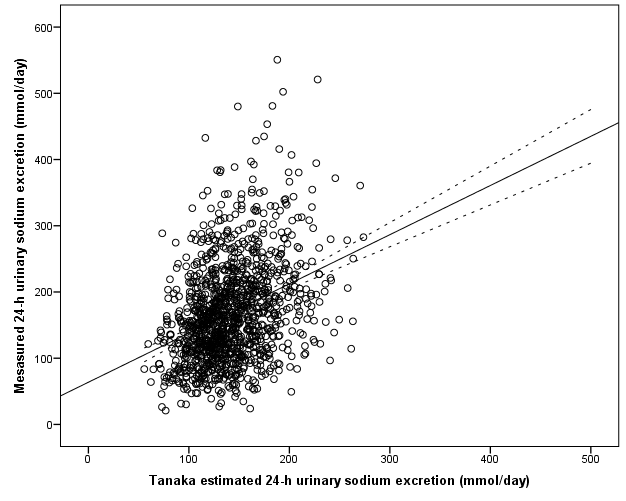


**C**

**A**

**B**

**r=0.36**

**r=0.35**

**r=0.36**

**F**

**E**

**D**


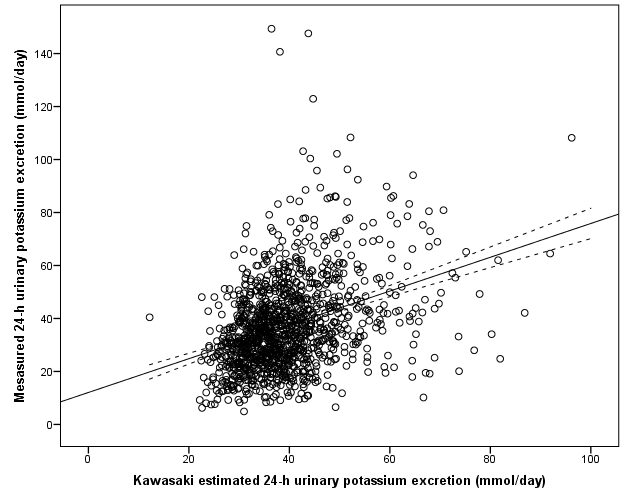

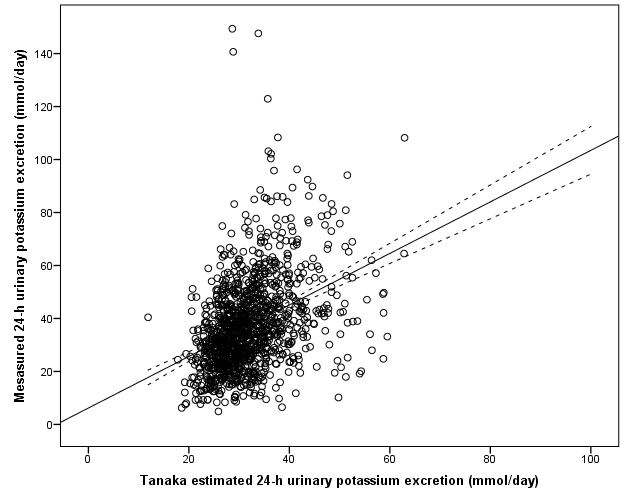

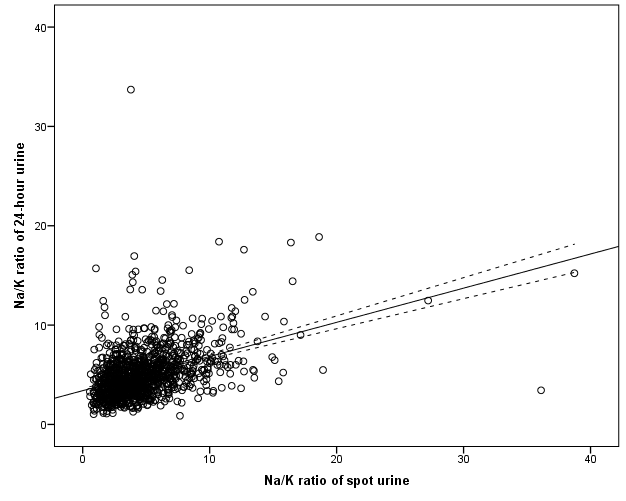


**r=0.42**

**r=0.38**

**r=0.36**

**Figure S2** Bland-Altman plots presenting measured vs. estimated 24-h UNa using the K-method (A), I-method (B) and T-method(C), 24-h UK using the K-method (D) and T-method(E), Na/K ratio of spot urine vs. 24-h urine (F). The mid-dashed line was the mean difference. The upper and lower limits of agreement was the mean difference±1.96×standard deviation. (1226 participants excluding antihypertensive medication).


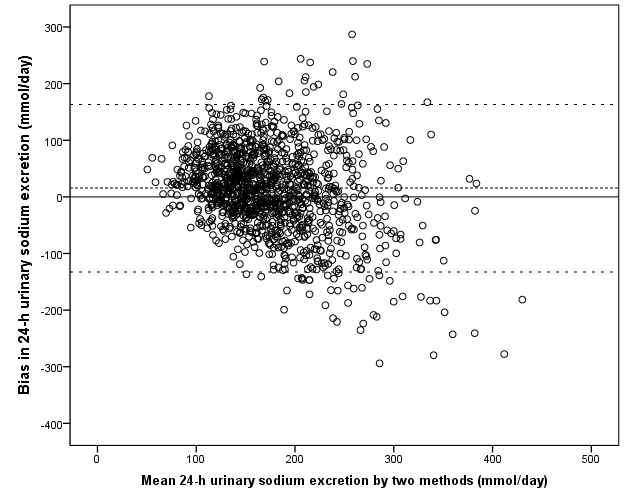

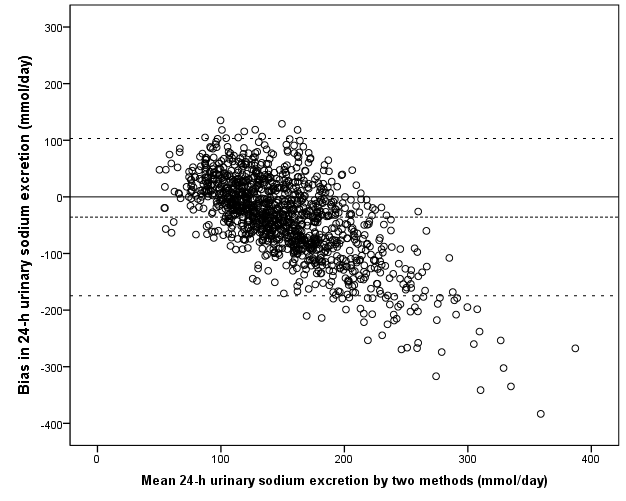

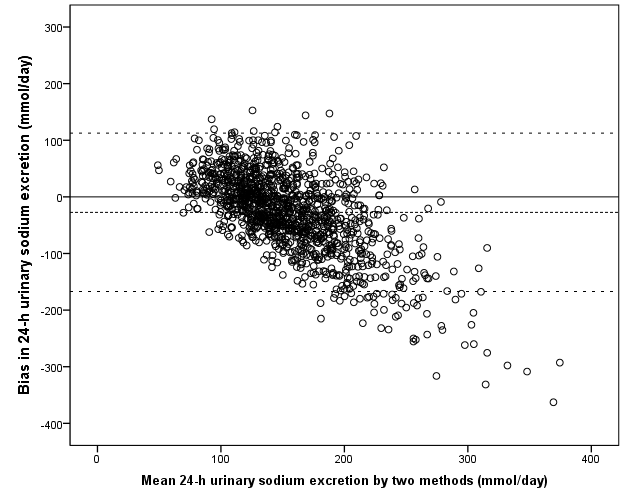


**C**

**B**

**A**

**Lower limit -167.24**

**Bias -27.35**

**Upper limit 112.54**

**Lower limit -174.75**

**Bias -35.88**

**Upper limit 102.99**

**Lower limit -135.74**

**Bias 13.65**

**Upper limit 163.04**

**F**

**E**

**D**


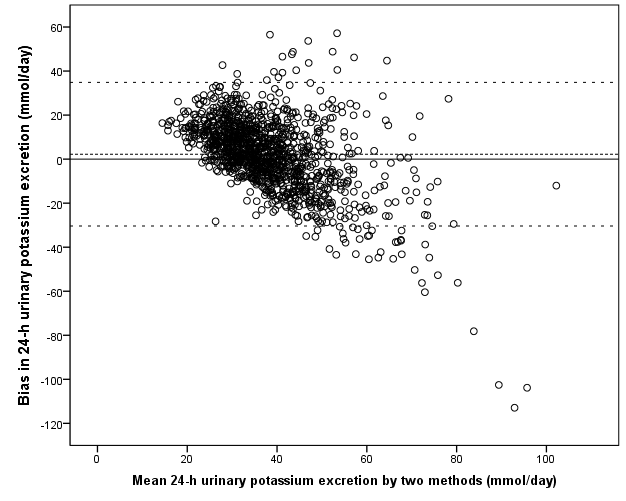

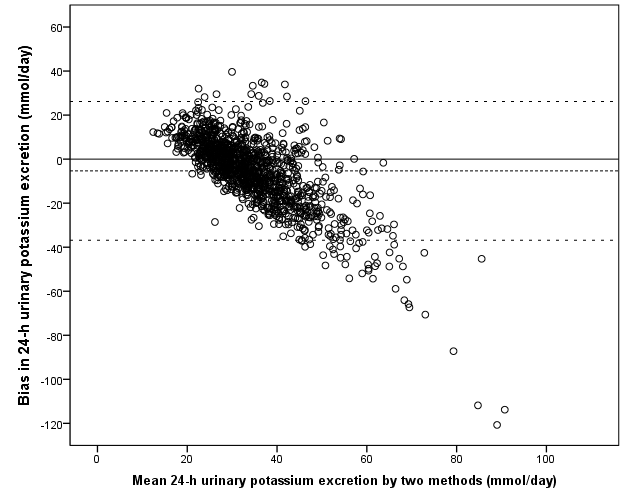

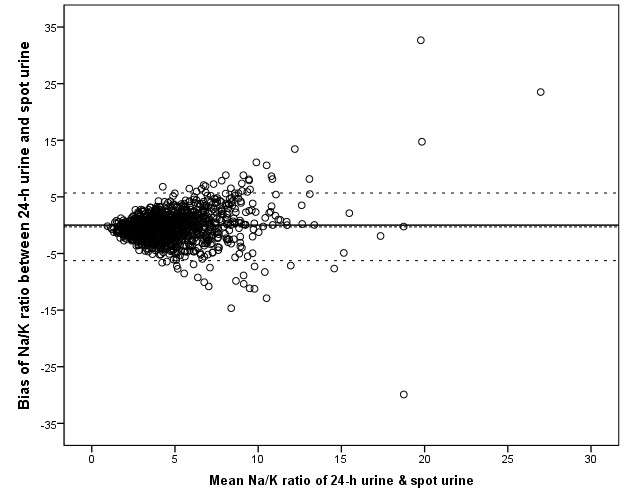


**Bias -5.34**

**Lower limit -6.27**

**Bias -0.29**

**Lower limit -36.86**

**Lower limit -30.38**

**Bias 2.23**

**Upper limit 5.69**

**Upper limit 26.18**

**Upper limit 34.84**
